# Clinical course and management of insidious adrenal crisis manifested initially as hyperpyrexia secondary to pembrolizumab: Case reports and literature review

**DOI:** 10.3389/fonc.2022.981084

**Published:** 2022-08-11

**Authors:** Dandan Geng, Yingnan Wang, Xin Zhang, Chenguang Zhao, Yao Fan, Chang Liu, Jinmei Wei, Bingjie Huo, Yang Zhao, Fengbin Zhang, Ruixing Zhang

**Affiliations:** ^1^ Department of Gastroenterology and Hepatology, The Fourth Hospital of Hebei Medical University, Shijiazhuang, China; ^2^ Department of Traditional Chinese Medicine, The Fourth Hospital of Hebei Medical University, Shijiazhuang, China; ^3^ Office of Academic Research, The Fourth Hospital of Hebei Medical University, Shijiazhuang, China

**Keywords:** pembrolizumab, immune-related adverse events (irAEs), adrenal crisis, immune checkpoint inhibitors (ICIs), hyperpyrexia

## Abstract

Immune checkpoint inhibitors (ICIs) are novel drugs with a dramatic survival benefit in patients with advanced malignancies. With the widespread use, several immune-related adverse events (irAEs) have emerged, which may be life-threatening. Herein we report two patients with adrenal crisis who received anti-programmed cell death protein 1 (PD-1) (pembrolizumab) therapy. Several reports of secondary adrenal insufficiency caused by pembrolizumab exist, including during treatment or late onset. Severe adrenal insufficiency according to the Common Terminology Criteria for Adverse Events (CTCAE) has rarely been described in the literature, since it initially manifests as high-grade fever. The two male patients developed adrenal crisis that was first characterized by hyperpyrexia accompanied by abdominal symptoms. These initial manifestations confused the clinicians who misdiagnosed them as infection. Timely identification, hydrocortisone pulse therapy, and fluid resuscitation improved the patients’ condition. Compliance with the standardized treatment approach and course can prevent or relieve the crisis as soon as possible. Assessment of relevant laboratory test results and patient education, including when to use stress-dose hydrocortisone and guidance on route of administration, can reduce the incidence of adrenal crisis. We report these two cases and have evaluated the literature on previously reported cases to improve our understanding of this condition and offer a more scientific approach to diagnosis and treatment options.

## Introduction

Cancer has so far been a major global public health problem, especially intractable malignant tumors (1). Nowadays, immune checkpoint inhibitors (ICIs) with surgery, chemotherapy, targeted therapy, and radiotherapy have become critical components of cancer treatment. ICIs, including anti-cytotoxic T lymphocyte-associated antigen 4 antibodies (CTLA-4), anti-programmed death receptor 1 (PD-1), and anti-PD-1 ligand (PD-L1), have proved to be effective against various malignancies since their initial Food and Drug Administration (FDA) approval in 2011 (2). Given the ever-expanding list of cancer types in which ICI has shown clinical effectiveness, the anti-oncologic spectrum is projected to rise in future. PD-1 regulates immune tolerance and prevents autoimmune responses in the physiological state by binding to its two ligands, PD-L1 and PD-L2, and this signaling aids tumor immunity evasion (3). Anti-PD-1 antibodies (for example, nivolumab and pembrolizumab) inhibit this immune-suppression pathway to reactivate T cell-mediated antitumor immunity and thus result in autoinflammation at other tissues while exerting their antitumor immunity effect. These clinically manifest as immune-related adverse events (irAEs). These irAEs are autoimmune in etiology and have varied onset and a wide range of toxicity involving the endocrine glands, gastrointestinal tract, skin, and liver, which are the most commonly involved organ systems (4, 5).

Endocrinopathy is among the most common irAEs associated with anti-PD-1 antibodies. The most common organ-specific endocrine irAE is hypothyroidism, while the occurrence of hypophysitis is rare (in <1% of cases) (6). Different from anti-CTLA-4 antibody-associated hypophysitis, presenting usually with multiple pituitary hormone deficiency, anti-PD-1 antibody-associated hypophysitis mostly involves the hypothalamic–pituitary–adrenal (HPA) axis, leading to secondary adrenal insufficiency (SAI). SAI is usually of grade 1–2 irAEs, while adrenal crisis (grade 4 irAE) is rare. Clinical manifestations of anti-PD-1-associated hypophysitis are usually non-specific and can be confused with tumor itself and chemotherapy side effects. Hypotension is the main feature of adrenal crisis, but it may be ignored when the clinical manifestations are atypical. Here, we report two cases of adrenal crisis secondary to pembrolizumab, presenting with hyperpyrexia as the initial clinical manifestation; we also conducted a literature review.

## Case description

### Case 1

**Figure 1:**
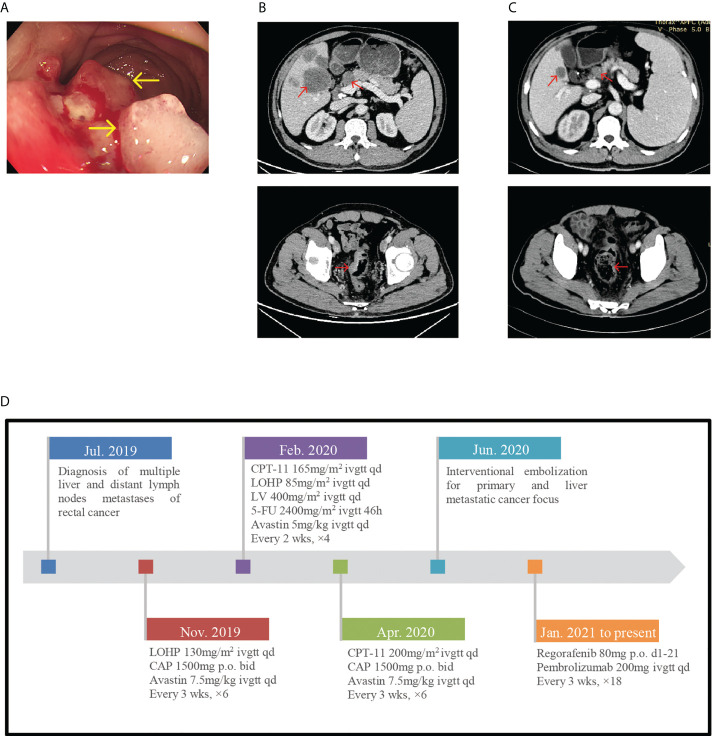
**(A)** Endoscope images showing rectal neoplasm. **(B)** In July 2019, pretreatment CT-scan showed the thickened colorectal wall and multiple liver and distant lymph nodes metastases. **(C)** CT-scan after 6 cycles of immunotherapy showed the tumor regression achieved partial response. **(D)** Timeline of diagnosis, detailed treatment protocol from July 2019 till date.

A 51-year-old man was admitted to the hospital with his electronic colonoscopy showing rectal neoplasm and pathological diagnosis revealing severe dysplasia of the glandular epithelium in July 2019 (**Figure 1A**). CT evaluation showed multiple liver and distant lymph node metastases (**Figure 1B**). A stage IV rectal cancer diagnosis was established. The patient had a history of third-stage hypertension for 15 years, and his usual blood pressure (BP) was maintained at approximately 145/90 mmHg. Furthermore, he developed type 2 diabetes for a year with poor control.

Since 4 July 2019, he was receiving antitumor treatment, including chemotherapy, targeted therapy, and interventional embolization for primary and liver metastatic cancer focus (**Figure 1D** shows detailed treatment protocol from July 2019 till date). With CT scan revealing disease development and pathological examination of colonoscopy whole exon gene showing tumor mutation burden (TMB) 34.12 Muts/Mb, he commenced chemotherapy treatment with regorafenib (80 mg orally d1-21, for 3 weeks) combined with pembrolizumab (200 mg injection d1 3 weeks) on 19 January 2021. After six cycles of pembrolizumab injection, the patient did not experience any discomfort and CT scan showed partial response (PR) (**Figure 1C**).

However, he presented to the emergency room on 4 July 2021, with hyperpyrexia, severe diarrhea, oliguria, and complaint of headache and blurred vision. Furthermore, he had a history of nausea and vomiting preadmission. His body temperature (BT) was 39.7°C, BP dropped to 114/83 mmHg, and facial expression was apathetic despite fluid resuscitation. Laboratory data revealed high creatinine level (175.2; normal: 57–97 μmol/l), hyponatremia (128; normal: 137–147 mmol/l), hyperglycemia (14.57; normal: 3.9–6.1 mmol/l), high procalcitonin level (2.640; normal 0.046 ng/ml), and normal leukocyte count (5.43 × 10^9^/l; normal: 3.5–9.5 × 10^9^/l). According to his clinical manifestations and laboratory results, the initial diagnosis was severe enterogenic infection. After positive fluid infusion, his creatinine and blood natremia levels normalized. Procalcitonin and C-reactive protein (CRP) levels resembled normal levels after empiric antibiotic therapy (meropenem and vancomycin). However, his BT remained high and BP was 125/75 mmHg despite sufficient fluid support and maintaining electrolyte balance. The blood glucose decreased to 5.57 mmol/l without hypoglycemic therapy. Given the history of immunotherapy, the physician considered another diagnosis. Further hormone test indicated normal thyroid function. However, at 8 a.m., the adrenocorticotropic hormone (ACTH) level was <1 pg/ml (normal: 7.2–63.3 pg/ml) and early-morning cortisol was 4.08 nmol/l (normal: a.m. (6:00–10:00) 133–537 nmol/l). The remaining anterior pituitary hormone levels were almost normal. Simultaneously, fecal bacterial culture before antibiotic therapy showed no remarkable result; this ruled out the enterogenic infection diagnosis. Consequently, considering the BP of <20 mmHg than usual, adrenal crisis induced by hypophysitis was the most plausible diagnosis, which is a grade 3–4 pembrolizumab-induced adverse reaction according to the Common Terminology Criteria for Adverse Events (CTCAE). Due to claustrophobia, pituitary magnetic resonance imaging (MRI) could not be performed. Subsequently, high-dose hydrocortisone i.v. drip (50 mg/day) was commenced, with continuous fluid resuscitation. Fever and diarrhea ceased, and other symptoms improved. Two days later, due to a rapid tapering off of hydrocortisone (20 and 10 mg orally in the morning and evening, respectively), the patient developed fever again (BT, 38.2°C). Oral hydrocortisone was discontinued quickly and replaced with 50 mg hydrocortisone i.v. drip daily for 4 days according to the endocrinologists. Symptoms significantly improved with BT and gradual BP normalization (146/79 mmHg) without antihypertensive therapy. Finally, the patient was safely discharged to outpatient endocrinology follow-up. Repeated testing still showed an undetectable ACTH level, consistent with the diagnosis, and the cortisol level normalized (396.1 nmol/l). Abdominal CT evaluation was maintained at PR (**Figure 1C**). With cortisol replacement therapy, pembrolizumab was reapplied on the patient on 19 August 2021 with continued use over the following treatment cycles. Moreover, there was no recurrence of adrenal crisis and other immune-related adverse during follow-up.

### Case 2

**Figure 2:**
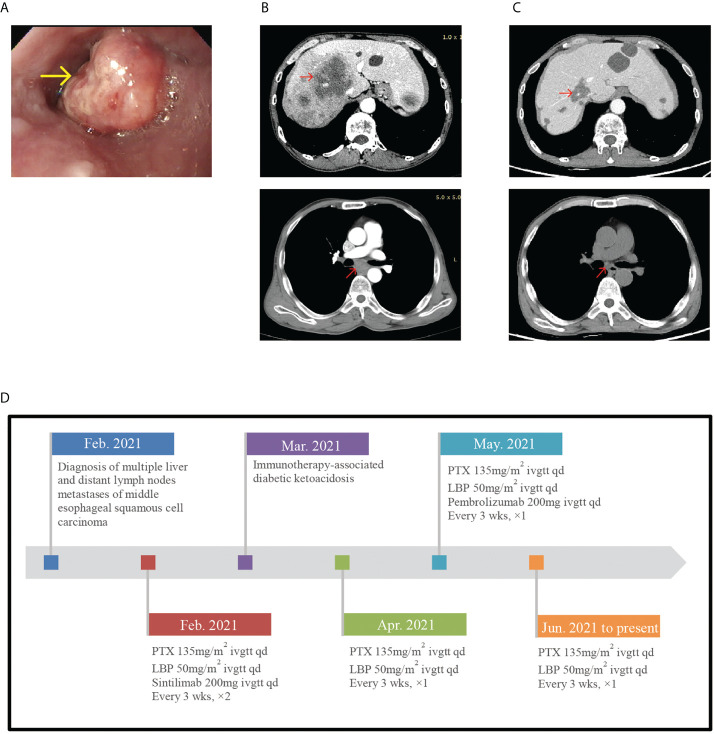
**(A)** Endoscope images showing esophageal neoplasm. **(B)** In February 2021, pretreatment CT-scan showed multiple livermetastases and supraclavicular and subcarinal lymph node metastases. **(C)** In June 2021, posttreatment CT-scan showed the tumor regression. **(D)** Timeline of diagnosis, detailed treatment protocols since February 2021 till date.

A 58-year-old man was diagnosed with stage IV (multiple liver metastases and supraclavicular and subcarinal lymph node metastases) middle esophageal squamous cell carcinoma in February 2021 (**Figures 2A, B** **)**. Based on the good tumor response and strong patient dependency on immunotherapy, the patient was treated with paclitaxel liposome (210-mg injection d1 3w), lobaplatin (60-mg injection d1 3w), and pembrolizumab (200-mg injection d1 3w) for one cycle. In May 2021, routine examination of the serum electrolyte outside the hospital revealed hyponatremia (129 mmol/l). His pituitary hormonal profile was unremarkable, except that the ACTH level was <1 pg/ml. Pembrolizumab-induced SAI was considered without any symptom. Therefore, he received prednisone (5 mg/day orally) as replacement therapy and pembrolizumab was discontinued for one cycle (**Figure 2D** shows detailed treatment protocols since February 2021 till date). Hormone reexamination showed a similar ACTH level as before and low levels of morning cortisol (27.80 nmol/l). His medication was adjusted to oral hydrocortisone (20 mg, morning; 10 mg, evening). The patient had a history of type 1 diabetes associated with ICI for almost 3 months with insulin therapy and poorly controlled glycemia.

In June 2021, the patient experienced nausea, vomiting, and diarrhea at home. A few days later, he presented to our inpatient department with complaints of hyperpyrexia and left abdominal pain. On physical examination, his BT was 39.9°C and BP level had decreased to 89/56 mmHg. Laboratory evaluation demonstrated normal serum sodium level (138.0 mmol/l) and leukocyte count (4.87 × 109/l), high procalcitonin (0.540 ng/ml), and high CRP (122.05 mg/l). Blood culture showed no bacterial growth. In addition, he developed hypoglycemia (2.63 mmol/l). The thyroid gland axis was unremarkable. His serum morning cortisol level was 83.72 nmol/l, his ACTH level was low (1.85 pg/ml), and his growth hormone (4679; normal: 30–2470 pg/ml), prolactin (17.96 ng/ml), and follicle-stimulating hormone (13.77; normal: 1.5–12.4 mIU/l) levels were high. No pituitary MRI was available for this patient. According to the symptoms and abnormal laboratory results, we considered pembrolizumab-induced severe SAI manifesting as adrenal crisis, a grade 3–4 pembrolizumab-induced adverse reaction according to the CTCAE. The patient was immediately injected with hydrocortisone sodium succinate (50 mg, q8h d1-3) with fluid resuscitation. After initiating glucocorticoid therapy, the patient became afebrile and his symptoms resolved swiftly with BP normalizing. His blood glucose level rose to 9.46 mmol/l. Procalcitonin and CRP levels were almost normal. The serum morning cortisol level rose to 586.50 nmol/l 3 days later, and we adjusted the hydrocortisone sodium succinate dose to 50 mg/day. In outpatient endocrinology, he received hydrocortisone acetate (20 and 10 mg orally in the morning and evening, respectively) as replacement therapy after discharge and CT scan evaluation showed no tumor progression (**Figure 2C**). During follow-up, this patient developed immune relevant hepatic damage and did not receive immune treatment anymore.

## Discussion

We presented two cases of adrenal crisis characterized by hyperpyrexia concurrent with abdominal symptoms following pembrolizumab as a combination therapy. This was a grade 3–4 endocrine irAE associated with anti-PD-1 antibodies. The non-specific symptoms made the central AI (CAI) diagnosis difficult, with clinicians misdiagnosing it as an infection. Noteworthily, a seemingly normal BP level could not rule out the crisis. Treatment using parenteral stress-dose hydrocortisone and intravenous rehydration with isotonic saline and subsequent tapering off to replacement-dose hydrocortisone is the main strategy. Because hydrocortisone was reduced too quickly, one patient re-presented with fever and a sufficient course of parenteral hydrocortisone finally reduced his crisis. Tumor imaging evaluation of both patients showed PR during follow-up; whether the occurrence of irAEs such as hypophysitis suggests a better clinical efficacy deserves further investigation.

Anti-PD-1 antibody-induced endocrine irAEs include thyroid dysfunction, adrenal insufficiency (AI), hypophysitis, and type 1 diabetes (7). CAI is the most common manifestation of hypophysitis because hypophysitis most often damages ACTH-producing cells (8) while those only affecting the HPA axis function are called isolated ACTH deficiency. This is most common with anti-PD-1, especially nivolumab and pembrolizumab. As the follow-up pituitary hormone results in the two patients showed, both patients developed isolated ACTH deficiency. Anti-PD1 agent-induced hypophysitis is usually of grade 1–2 irAEs while grade 3–4 irAEs, especially adrenal crisis, are rarely reported. According to Barroso-Sousa et al., grade ≥3 hypophysitis was reported in 34 (0.5%) cases (6), and in a large study including 125 clinical trials and 20,128 patients, the most frequent endocrine irAE is hypothyroidism (6.07%), while hypophysitis is <1% (0.60%). In grade ≥3 irAEs, hypophysitis (0.16%) is rare, but with higher occurrence than hypothyroidism (0.08%) (grade ≥3 irAE risk ratio, 26.67% *vs*. 1.32%, respectively) (7). The contradiction between incidence rate and risk ratio resulted in part from the frequent monitoring of the thyroid-stimulating hormone (TSH) in the clinic, with early detection of thyroid dysfunction. However, due to the relatively low hypophysitis incidence, routine monitoring of adrenal and pituitary function is not yet prevalent in clinical practice. Yano et al. reported that human leucocyte antigen, DR15, might predict ICI-associated SAI onset, while no definitive predictive biomarkers have been discovered (9). Thus, high vigilance and routine detection in clinical practice is essential.

The main clinical manifestations of adrenal crisis are severe hypotension or hypovolemic shock, acute abdomen, vomiting, high fever or hypothermia, and hypoglycemic attack. Among these, hypotension is the core symptom in adrenal crisis diagnosis. Our first patient presented with high fever and diarrhea while the second presented with high fever and abdominal pain. Hypotension and other symptoms were not obvious at the early stage, and along with increased procalcitonin and CRP levels, infection could be misdiagnosed. While anti-infection treatment did not improve their symptoms significantly, their condition progressed to a critical state. The first patient developed relative hypotension with SBP ≥30 mmHg lower than usual, along with an elevated serum creatinine level, indicating insufficient blood volume. The second patient developed absolute hypotension, until hormone supplementation improved his condition. Therefore, in-depth understanding of adrenal crisis and early diagnosis and treatment are of great clinical importance (10). Meanwhile, unexplained hyponatremia should always be considered in diagnosing AI (11). Oncologists who use immunotherapy acknowledge this. However, in both patients, mild hyponatremia was ignored due to diarrhea and insufficient food intake. Therefore, for patients with hyponatremia and high fever, it is still necessary for clinicians to be vigilant, to timeously identify incipient adrenal crisis, for early prevention. Particularly, with abdominal symptoms, erroneous diagnosis of gastroenteritis may delay hormone therapy.

For diagnosing AI, along with the corresponding laboratory examinations, pituitary MRI, which is not essential for diagnosis AI promptly, may also be positive. PD-1 inhibitor-associated hypophysitis usually presents with a normal pituitary gland image on MRI, while CTLA-4 inhibitor-related hypophysitis more commonly shows pituitary enlargement (12–15). Oğuz et al. reported a rare case of pembrolizumab-associated isolated ACTH deficiency with mildly enlarged pituitary MRI (15). Therefore, when endocrinological examination indicates CAI, hormone treatment should not be delayed to await pituitary MRI. In the 51-year-old patient, headache and blurred vision disappeared after hydrocortisone pulse therapy, which can rule out secondary adrenal insufficiency (SAH) by other causes (most commonly, pituitary and adjacent tissue tumors) (11).

Many scholars propose that even if grade 3–4 endocrine irAEs occur, immunotherapy can be restored when the patient is stabilized on replacement hormones (6, 8, 16). Because of its physiological glucocorticoid pharmacokinetics, plasma protein binding, tissue distribution, and balanced glucocorticoid-mineralocorticoid actions, hydrocortisone is the ideal medicine for treating adrenal crisis (10, 11). Due to insufficient dose of pulse therapy, our patients’ BP did not rise within 1 h as stated in the definition of adrenal crisis (10) but gradually increased to the pre-crisis level. When immunotherapy-associated adrenal crisis happens, besides supportive fluid therapy, initial intravenous or intramuscular bolus injection of 100 mg hydrocortisone is required, along with continuous intravenous infusion of 200 mg hydrocortisone q24h (daily) or, alternatively, intravenous or intramuscular bolus injections of 50 mg hydrocortisone q6h (or as 50 mg four times daily) (10). The recommended duration is 24–48 h until the patient can receive oral hydrocortisone (17). Then, the hydrocortisone dose should be reduced in accordance with the patient’s clinical response, with BP and clinical symptoms closely monitored. Of note, doctors should be careful of symptoms of recurrence while reducing the hormone dose. Just like our patient, after reducing the dose of hydrocortisone, the patient developed fever again. Moreover, his condition improved until the dose was maintained at 50 mg by iv. drip for 4 days.

This case revealed that patients generally benefit from adequate therapy course and a slower dose reduction course of hormone. Three days are usually recommended to reduce the hydrocortisone dose to the maintenance level (10). When the patient’s condition is stable, glucocorticoid replacement should be the mainstay treatment. Patients should use medicine for a long time, or permanently, because many studies have shown that the pituitary–adrenal axis damage is irreversible, different from thyrotropin deficiency that is much more likely to reverse (18, 19). One explanation is that immunological activity destroys practically all hormone-producing cells, preventing the recovery of hormone production; however, histological evidence is lacking. Levy et al. (20) described one case of recovery of ACTH deficiency induced by pembrolizumab, which presented as grade 1 hypophysitis. Faje et al. and Ryder et al. also described cases of recovery of normal ACTH function induced by ipilimumab or combination therapy (14, 21, 22). Whether the pituitary–adrenal axis function can recover remains unknown; however, as suggested, regular monitoring of 8 a.m. cortisol before using hydrocortisone may be essential for ACTH recovery.

For an adult, an oral maintenance dose of hydrocortisone is required to be 15–25 mg per day (11). Clinicians have different views on the frequency and dose of hydrocortisone. Husebye et al. recommend that the amount of hydrocortisone should not exceed 10 mg each time, usually two to four times daily, because an increased area under the curve of hydrocortisone is not directly proportional to an increased maximum blood drug concentration. If the one-time dose exceeds 10 mg, the corresponding blood drug concentration will not increase accordingly (11). Thus, modified-release hydrocortisone taken once daily (15–25 mg) or *via* subcutaneous pump treatment is also recommended for patients trying to optimize traditional treatments. However, in most cases, the amount of each replacement treatment generally exceeds 10 mg; thus, the optimal administration scheme is still worth exploring. Significantly, when suffering from stress, the patients’ glucocorticoid doses should be increased or they should switch to parenteral injections; these play a key role in averting the rapid condition deterioration (11). Treatment should continue with oral glucocorticoids at doubled or tripled replacement doses until stress relief, then the daily maintenance dose is resumed (3). In our case 2, although SAI was diagnosed early and treated with hormone, when the patient developed stress events (vomiting and diarrhea), the dose was not increased in time, resulting in adrenal crisis. Some scholars indicate that when patients have gastrointestinal symptoms, a timely administration of parenteral glucocorticoids is essential, because vomiting and diarrhea may impair the absorption of oral glucocorticoids (23). Of note, serum electrolyte and BP monitoring rather than serum cortisol and ACTH levels are indicators of appropriate glucocorticoid replacement. Low serum sodium level and postural hypotension are the main manifestations of glucocorticoid deficiency. The electrolyte level, BP, and blood glucose level in our patients returned to normal after hormone treatment, and the clinical symptoms disappeared; these reflect the effect of replacement therapy.

The robust and aggressive immune recruitment induced by ICIs leading to rapid and complete tissue destruction is more rapid than the classic autoimmune diseases (24). Many people experience adrenal crisis before being diagnosed with AI. Therefore, prompt identification and treatment initiation for the condition before a crisis occurs can have dramatic effects on the patients’ health and quality of life. Because the initial clinical manifestations can be atypical, they may be ignored; therefore, regular assessment is essential including fasting venous glycemia, natremia, TSH, free T4, 8 a.m. cortisol (without corticosteroid intake), ACTH, LH, FSH, testosterone in men, and FSH in menopausal women, and subjective symptoms (25, 26). For better management, physicians should educate patients on the use of oral stress dosing of hydrocortisone and parenteral hydrocortisone administration when required (11, 17). In a stress state (with vomiting or severe illness), parenteral hydrocortisone can be administered intramuscularly or subcutaneously, outside the hospital, while rectal hydrocortisone suppositories can be used as an alternative in some circumstances. Sonehara et al. (27), Boudjemaa et al. (28), and Yamagata et al. (29) reported late-onset SAI after pembrolizumab, which revealed a favorable prognostic factor associated with survival just as our patients showed non-progression at follow-up efficacy evaluation. On the other hand, this suggests that irAEs may also occur even after pembrolizumab withdrawal and require a high index of suspicion for a timely diagnosis.

## Data availability statement

The original contributions presented in the study are included in the article/supplementary material. Further inquiries can be directed to the corresponding authors.

## Author contributions

DG and YW contributed to study concepts, study design, data acquisition, interpretation, manuscript preparation, manuscript editing, and manuscript review. XZ, CZ, YF, and CL contributed to study concepts, study design, manuscript editing, and manuscript review. JW, BH, and YZ contributed to manuscript editing and manuscript review. FZ and RZ contributed to study concepts, study design, data acquisition, quality control of data, data analysis and interpretation, manuscript editing, and manuscript review. All authors contributed to the article and approved the submitted version.

## Funding

This study was funded by the Key Research and Development Projects of Hebei Province (Grant no. 19277736D).

## Acknowledgments

The authors would like to thank the patients who consented to participate and release their personal data for the scientific purposes of this research.

## Conflict of interest

The authors declare that the research was conducted in the absence of any commercial or financial relationships that could be construed as a potential conflict of interest.

## Publisher’s note

All claims expressed in this article are solely those of the authors and do not necessarily represent those of their affiliated organizations, or those of the publisher, the editors and the reviewers. Any product that may be evaluated in this article, or claim that may be made by its manufacturer, is not guaranteed or endorsed by the publisher.
